# Supplementary material for: A multicentric consortium study demonstrates that dimethylarginine dimethylaminohydrolase 2 is not a dimethylarginine dimethylaminohydrolase
Source: Nat Commun. 2023 Jun 9;14:3392. doi: 10.1038/s41467-023-38467-9 (PMC10256801; doi:10.1038/s41467-023-38467-9)
Supplement: Supplementary file 1 — Supplementary Information [file 41467_2023_38467_MOESM1_ESM.pdf]

Title

A multicentric consortium study demonstrates that dimethylarginine dimethylaminohydrolase 2 is not a dimethylarginine dimethylaminohydrolase

Authors

Vinitha N. Ragavan[1,2,22], Pramod C. Nair[2,3,4,5,22], Natalia Jarzebska[1], Ramcharan Singh Angom[6], Luana Ruta[7], Elisa Bianconi[7], Silvia Grottelli[8], Natalia D. Tararova[9], Daniel Ryazanskiy[9], Steven R. Lentz[10], Sara Tommasi[2], Jens Martens-Lobenhoffer[11], Toshiko Suzuki-Yamamoto[12], Masumi Kimoto[12], Elena Rubets[1], Sarah Chau[13], Yingjie Chen[14], Xinli Hu[15] Nadine Bernhardt[16], Peter M. Spieth[17], Norbert Weiss[1], Stefan R. Bornstein[1,18], Debabrata Mukhopadhyay[6], Stefanie M. Bode-Böger[11], Renke Maas[19,20], Ying Wang[13], Antonio Macchiarulo[7], Arduino A. Mangoni[2], Barbara Cellini[8], Roman N. Rodionov[1,21*]

[1]Department of Internal Medicine III, Technische Universität Dresden, Dresden, Germany.

[2]Department of Clinical Pharmacology, College of Medicine and Public Health, Flinders University and Flinders Medical Centre, Bedford Park, Adelaide, SA, Australia.

[3]Flinders Health and Medical Research Institute (FHMRI), College of Medicine and Public Health, Flinders University, Adelaide, SA, Australia.

[4]Cancer Program, South Australian Health and Medical Research Institute (SAHMRI), University of Adelaide, Adelaide, SA, Australia.

[5]Discipline of Medicine, Adelaide Medical School, The University of Adelaide, Adelaide, SA, Australia.

[6]Department of Biochemistry and Molecular Biology, Mayo Clinic College of Medicine and Science, Jacksonville, FL, USA.

[7]Department of Pharmaceutical Sciences, University of Perugia, via del Liceo 1, Perugia, Italy.

[8]Department of Medicine and Surgery, University of Perugia, P.le L. Sevari 1, Perugia, Italy.

[9]Dapcel, Inc. Cleveland, OH, USA.

[10]Department of Internal Medicine, The University of Iowa Carver College of Medicine, Iowa City, IA, USA.

[11]Institute of Clinical Pharmacology, Otto von Guericke University, Magdeburg, Germany.

[12]Department of Nutritional Science, Faculty of Health and Welfare Science, Okayama Prefectural University, Okayama, Japan.

[13]Department of Cardiovascular Medicine, Mayo Clinic College of Medicine and Science, Rochester, NY, USA.

[14]Department of Physiology and Biophysics, University of Mississippi Medical Center, Jackson, NY, USA.

[15]Institute of Molecular Medicine, Beijing University, Beijing, China.

[16]Department of Psychiatry and Psychotherapy, University Hospital Carl Gustav Carus, Technische Universität Dresden, Dresden, Germany.

[17]Department of Anesthesiology and Critical Care Medicine, University Hospital Dresden, Technische Universität Dresden, Dresden, Germany.

[18]School of Cardiovascular and Metabolic Medicine and Sciences, Faculty of Life Sciences & Medicine, King's College London, London, UK.

[19]Institute of Experimental and Clinical Pharmacology and Toxicology, Friedrich-Alexander-Universität Erlangen-Nürnberg, Erlangen, Germany.

[20]FAU New – Research Center for New Bioactive Compounds, Friedrich-Alexander-Universität Erlangen-Nürnberg, Erlangen, Germany.

[21]College of Medicine and Public Health, Flinders University and Flinders Medical Centre, Adelaide, SA, Australia.

[22]These authors contributed equally

* Corresponding author

Roman N. Rodionov, MD, PhD, FAHA

University Center for Vascular Medicine

University Hospital Carl Gustav Carus, Technische Universität Dresden

Fetscherstrasse 74, 01307 Dresden, Germany

Phone: +49-351-458-3659

Fax: +49-351-458-4359

E-Mail: Roman.Rodionov@uniklinikum-dresden.de

Supplementary Figures

```
#=======================================
#
# Aligned_sequences: 2
# 1: NP_036269.1
# 2: NP_001289936.1
# Matrix: EBLOSUM62
# Gap_penalty: 10.0
# Extend_penalty: 0.5
#
# Length: 289
# Identity:     145/289 (50.2%)
# Similarity:   193/289 (66.8%)
# Gaps:           8/289 ( 2.8%)
# Score: 685.5
#
#
#=======================================

NP_036269.1        1 MAGLGHPA-AFGRATHAVVRALPESLGQHALRSAKGEEVDVARAERQHQL     49
                        :|.|. ..||.:||::|.:||||.......|....:|:|:|:|:|.:
NP_001289936.      1 ---MGTPGEGLGRCSHALIRGVPESLASGEGAGAGLPALDLAKAQREHGV     47

NP_036269.1       50 YVGVLGSKLGLQVVELPADESLPDCVFVEDVAVVCEETALITRPGAPSRR     99
                     ..|.|..:||||::|||.:||||....:.|.||:..:|||||||.:|:||
NP_001289936.     48 LGGKLRQRLGLQLLELPPEESLPLGPLLGDTAVIQGDTALITRPWSPARR     97

NP_036269.1      100 KEVDMMKEALEKLQLNIVEMKDENATLDGGDVLFTGREFFVGLSKRTNQR    149
                     .|||.:::||:.|.|.|||:.||||||||.|||||||||||||||.||.|
NP_001289936.     98 PEVDGVRKALQDLGLRIVEIGDENATLDGTDVLFTGREFFVGLSKWTNHR    147

NP_036269.1      150 GAEILADTFKDYAVSTVPVADGLHLKSFCSMAGPNLIAIGSSESAQKALK    199
                     ||||:||||:|:|||||||:...||:..|.|.||..:..|||::||||::
NP_001289936.    148 GAEIVADTFRDFAVSTVPVSGPSHLRGLCGMGGPRTVVAGSSDAAQKAVR    197

NP_036269.1      200 IMQQMSDHRYDKLTVPDDIAANCIYL--NIPNKGHVLLHRTPEEYPESAK    247
                     .|..::||.|..||:|||.||:|::|  .:|.....||||...:.|.|.:
NP_001289936.    198 AMAVLTDHPYASLTLPDDAAADCLFLRPGLPGVPPFLLHRGGGDLPNSQE    247

NP_036269.1      248 VYEKLKDHMLIPVSMSELEKVD-GLLTCCSVLINKKVDS    285
                     ..:||.|..|:|||.|||||.. ||.:.|.||..:...
NP_001289936.    248 ALQKLSDVTLVPVSCSELEKAGAGLSSLCLVLSTRPHS-    285
```

Supplementary Figure 1. Amino acid alignment of human DDAH1 and DDAH2 sequences. The amino acid sequences of DDAH1 and DDAH2 from human were aligned using the Needle Pairwise Sequence Alignment tool by EMBOSS Programs. A space identifies mismatch or gaps, a period identifies a small positive score, a colon identifies a similarity with a score of more than 1.0 while a perpendicular line identifies sequence identity. NP_036269.1 = Homo sapiens DDAH1; NP_001289936.1 = Homo sapiens DDAH2.

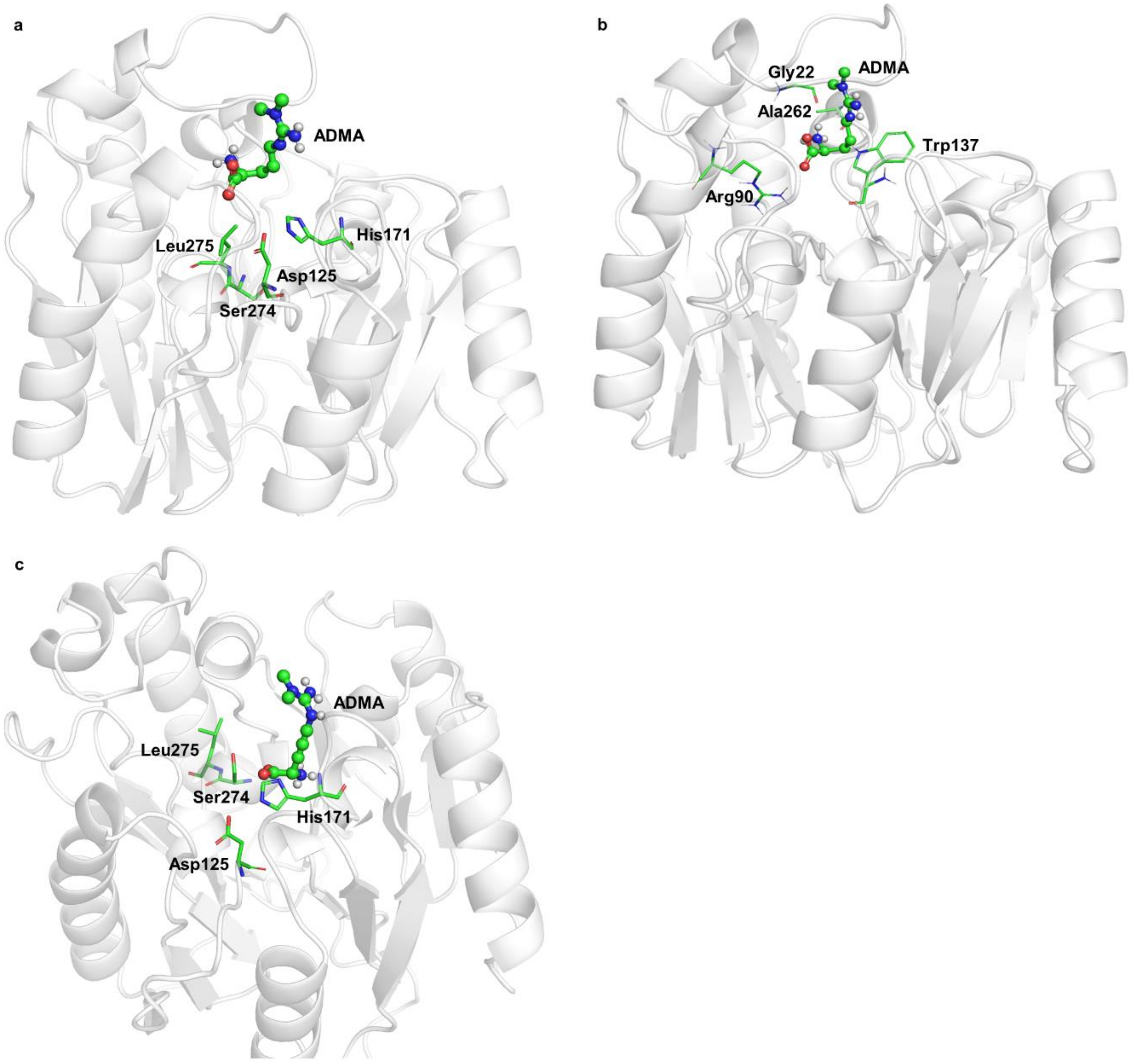

Supplementary Figure 2. Binding mode of ADMA in DDAH2 structures using molecular docking. Molecular docking of ADMA in DDAH2 Model A (SWISS-MODEL) using SYBYL (X-2.1), with **a** residues of the putative binding site and **b** interacting residues outside the

putative binding site. **c** Blind docking of ADMA in DDAH2 Model B (AlphaFold) using Flare (V6.1), with residues of the putative binding site highlighted. ADMA is shown in ball and stick, key residues are shown in sticks, and DDAH2 structures are shown as cartoon (white). C, O, N are shown in green, red, and blue, respectively. Source data are provided as a Source Data file.

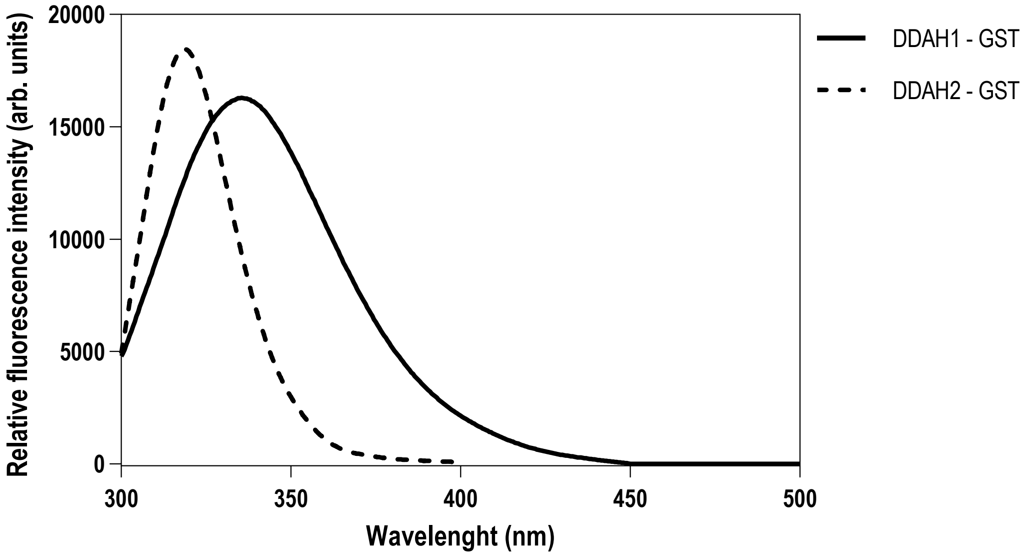

Supplementary Figure 3. Intrinsic fluorescence spectra of recombinant DDAH proteins. DDAH1-GST (straight line) and DDAH2-GST (dashed line) fluorescence spectra in 20 mM Tris-HCl, 150 mM NaCl, pH 8.5 at 1 mg/ml protein concentration were acquired. Proteins were excited at 280nm. arb. units = arbitrary units. Source data are provided as a Source Data file.

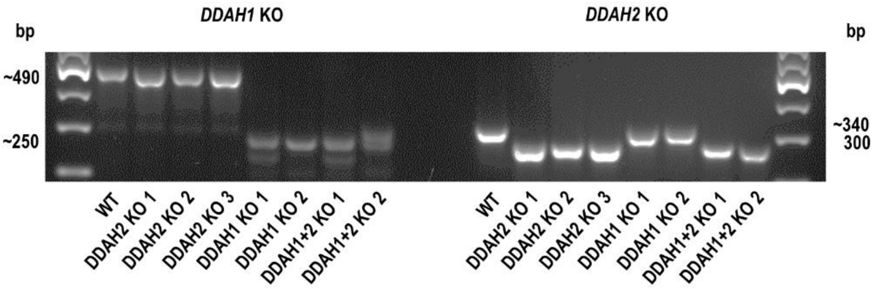

Supplementary Figure 4. Genotyping PCR results of CRISPR Cas9-mediated *DDAH* knockout MDA-MB-231 clones. Cell lines were genotyped using DDAH1 KO and DDAH2

KO primer pairs to confirm the CRISPR Cas9-mediated deletion at the respective target sites. bp = base pair. Source data are provided as a Source Data file.

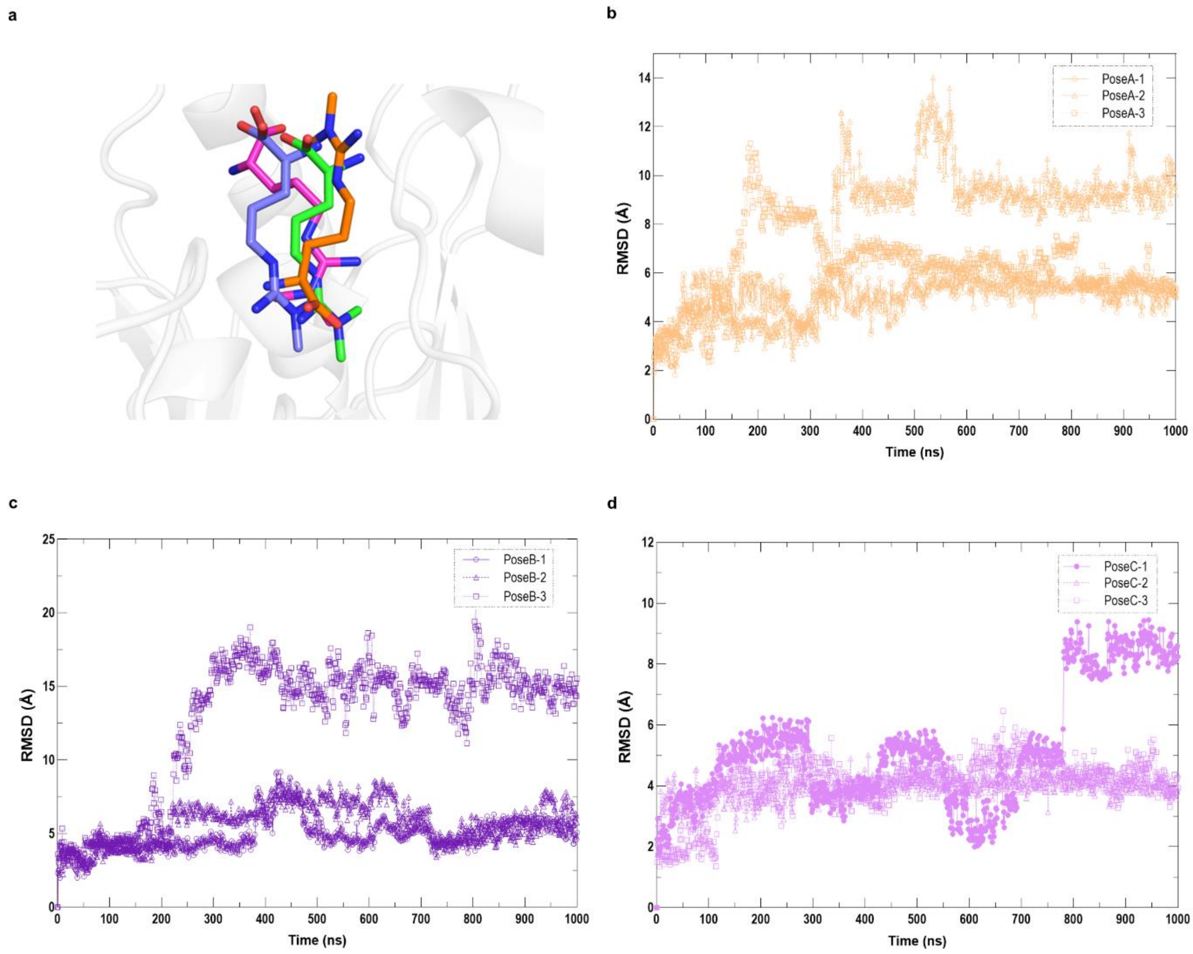

Supplementary Figure 5. Molecular dynamics simulations of ADMA (different binding poses) in the DDAH2 (Model A) binding site. a Binding modes of ADMA, reference conformation ADMA (C-atoms in green, see Fig. 2) based on the citrulline conformation (2JAI), pose A (C-atoms orange), pose B (C-atoms purple), and pose C (C-atoms magenta). **b** Root mean square deviation (RMSD) of ADMA in pose A bound to the DDAH2 structure (mean RMSD ± SD of individual simulation in the triplicate run = 5.02 ± 0.78 Å, 7.72 ± 2.84 Å, 6.26 ± 1.44 Å). **c** RMSD of ADMA in pose B bound to the DDAH2 structure (mean RMSD ± SD of individual simulation in the triplicate run = 4.92 ± 1.10 Å, 5.73 ± 1.43 Å, 12.74 ± 4.62 Å). **d** RMSD of ADMA in pose C bound to the DDAH2 structure (mean RMSD ± SD of

individual simulation in the triplicate run = 5.21 ± 1.93 Å, 3.95 ± 0.40 Å, 4.07 ± 0.92 Å). Source data are provided as a Source Data file.

Supplementary Tables

Supplementary Table 1. Mean of root mean square deviation (RMSD) (Å) of ADMA bound to DDAH structures from independent MD (Molecular Dynamics) simulation runs.

Simulations	DDAH1-ADMA MD Mean RMSD ± SD (Å)	DDAH2 (Model A)-ADMA MD Mean RMSD ± SD (Å)	DDAH2 (Model B)-ADMA MD Mean RMSD ± SD (Å)
Sim. 1	1.70 ± 0.13	7.69 ± 2.21	3.44 ± 0.56
Sim. 2	1.48 ± 0.14	4.67 ± 1.14	3.79 ± 1.06
Sim. 3	1.42 ± 0.33	5.86 ± 1.13	3.71 ± 0.59
Sim. 4	1.54 ± 0.27	3.84 ± 1.34	3.86 ± 1.03
Sim. 5	1.52 ± 0.15	4.11 ± 0.70	4.24 ± 0.86
Mean Sim. 1-5	1.53 ± 0.24	5.23 ± 1.99	3.81 ± 0.88
*p-value	-	****p<0.0001 (0.000077)	**p<0.01 (0.002179)

*Statistical analysis was performed using one-way ANOVA, with multiple comparisons to DDAH1-ADMA MD simulation runs (n = 5). The mean of each simulation was calculated from snapshot frames 1-1000, with frame 0 (0-100 ps) excluded to account for the equilibration phase of the simulations. Data are presented as mean ± SD. Sim. = simulation.

Supplementary Table 2: Primer list for quantitative polymerase chain reaction (qPCR) analysis.

Genes – Models	Primers
Human *ACTB* – HEK293T cell lines	F 5’-CTTCGCGGGCGACGAT-3’<br>R 5’-CCACATAGGAATCCTTCTGACC-3’
Human *ACTB* – HUVEC cell lines	F 5’-CCAACCGCGAGAAGATGA-3’<br>R 5’-CCAGAGGCGTACAGGATAG-3’
Human *HPRT* – MDA-MB-231 cell lines	F 5’-TTGCGACCTTGACCATCTTTG-3’<br>R 5’-CTTTGCTGACCTGCTGGATTAC-3’
Human *DDAH1* - HEK293T and MDA-MB-231 cell lines	F 5’-ATGCAACTTTAGATGGCGGAG-3‘<br>R 5’-CAGCCAAGATTTCAGCACCTC-3’
Human *DDAH2* - HEK293T and MDA-MB-231 cell lines	F 5’-CCACCTGAGGAGTCATTGCCGC-3’<br>R 5’-CGTGATTAGGGCCGTGTCCCCTT-3’
Human *DDAH1* - HUVEC cell lines	F 5‘-GTGCCCACTCCTGTTGTTTT-3‘<br>R 5‘-GGGGTGTTGAATGAAGCAAT-3‘
Human *DDAH2* - HUVEC cell lines	F 5‘-GGACTCCCTTCTCCACCAA-3‘<br>R 5‘-TTCTTGTTTCTTCACCTGTCTCC-3‘
Mouse *Actb*	F 5’-ACTGTCGAGTCGCGTCCA-3’<br>R 5-ATCCATGGCGAACTGGTGG-3’
Mouse *Ddah1* - $Ddah1^{-/-}$ and $Ddah2^{-/-}$ colonies	F 5’-CTACGCAGTCTCTACAGT-3’<br>R 5’-TCATAACGATGGTCACTCA-3’
Mouse *Ddah2* - $Ddah1^{-/-}$ colony	F 5’-AAAGCAGTCAGGGCAATG-3’<br>R 5’-CCAGGACGCAGAAAGAGA-3’
Mouse *Ddah2* - $Ddah2^{-/-}$ colony	F 5’- TCACTGCCGCTGGGACCACT-3<br>R 5’-CTCGCTGCTTCCAGCCACCA-3
