# Supplementary material for: A multicentric consortium study demonstrates that dimethylarginine dimethylaminohydrolase 2 is not a dimethylarginine dimethylaminohydrolase
Source: Nat Commun. 2023 Jun 9;14:3392. doi: 10.1038/s41467-023-38467-9 (PMC10256801; doi:10.1038/s41467-023-38467-9)
Supplement: Supplementary file 3 — Description of Additional Supplementary Files [file 41467_2023_38467_MOESM3_ESM.docx]

# Title

A multicentric consortium study demonstrates that dimethylarginine dimethylaminohydrolase 2 is not a dimethylarginine dimethylaminohydrolase

# Supplementary Movie Files Description

File Name: Supplementary Movie 1.

Description: Molecular dynamics simulation (MDS) of DDAH1-ADMA complex. MDS display a stable binding interaction between ADMA (atoms in sphere) and DDAH1 (cartoon) as reported in the X-ray structure.

File Name: Supplementary Movie 2

Description: Molecular dynamics simulation (MDS) of DDAH2-ADMA complex. MDS detected an unstable binding interaction between ADMA (atoms in sphere) and DDAH2 Model A (SWISS-MODEL) (cartoon).

File Name: Supplementary Movie 3

Description: Molecular dynamics simulation (MDS) of DDAH2-ADMA complex. MDS detected an unstable binding interaction between ADMA (atoms in sphere) and DDAH2 Model B (AlphaFold) (cartoon).

File Name: Supplementary Movie 4

Description: Molecular dynamics simulation (MDS) of DDAH2. Flexible loop within the DDAH2 (Model A, cartoon) binding site. Ser274 and Leu275 are shown in sticks. C, O, and N atoms are shown in green, red, and blue, respectively.
