# Supplementary material for: A multicentric consortium study demonstrates that dimethylarginine dimethylaminohydrolase 2 is not a dimethylarginine dimethylaminohydrolase
Source: Nat Commun. 2023 Jun 9;14:3392. doi: 10.1038/s41467-023-38467-9 (PMC10256801; doi:10.1038/s41467-023-38467-9)
Supplement: Supplementary file 8 — Reporting Summary [file 41467_2023_38467_MOESM8_ESM.pdf]

nature portfolio

Corresponding author(s): Roman N. Rodionov

Last updated by author(s): Apr 7, 2023

Reporting Summary

Nature Portfolio wishes to improve the reproducibility of the work that we publish. This form provides structure for consistency and transparency in reporting. For further information on Nature Portfolio policies, see our Editorial Policies and the Editorial Policy Checklist.

Statistics

For all statistical analyses, confirm that the following items are present in the figure legend, table legend, main text, or Methods section.

n/a	Confirmed	
[ ]	[x]	The exact sample size (*n*) for each experimental group/condition, given as a discrete number and unit of measurement
[ ]	[x]	A statement on whether measurements were taken from distinct samples or whether the same sample was measured repeatedly
[ ]	[x]	The statistical test(s) used AND whether they are one- or two-sided<br>*Only common tests should be described solely by name; describe more complex techniques in the Methods section.*
[x]	[ ]	A description of all covariates tested
[ ]	[x]	A description of any assumptions or corrections, such as tests of normality and adjustment for multiple comparisons
[ ]	[x]	A full description of the statistical parameters including central tendency (e.g. means) or other basic estimates (e.g. regression coefficient) AND variation (e.g. standard deviation) or associated estimates of uncertainty (e.g. confidence intervals)
[ ]	[x]	For null hypothesis testing, the test statistic (e.g. *F*, *t*, *r*) with confidence intervals, effect sizes, degrees of freedom and *P* value noted<br>*Give P values as exact values whenever suitable.*
[x]	[ ]	For Bayesian analysis, information on the choice of priors and Markov chain Monte Carlo settings
[x]	[ ]	For hierarchical and complex designs, identification of the appropriate level for tests and full reporting of outcomes
[x]	[ ]	Estimates of effect sizes (e.g. Cohen's *d*, Pearson's *r*), indicating how they were calculated

Our web collection on statistics for biologists contains articles on many of the points above.

Software and code

Policy information about availability of computer code

Data collection	Computational work used the following softwares: SWISS-MODEL (ref. 65), Alpha Fold (ref. 66-67), SYBYL (version X-2.1), Surflex-Doc (ref. 72), GROMACS 2020 (ref. 75-76), ChemAxon (Marvin 16.6.20), ACPYPE (ref. 77), Flare (V6.1), and PyMol (ref. 44). GuideRNA design was performed using the software Geneious 8.1.6. Sequencing results were analysed using Benchling. Western blot and agarose gel images were collected using PeqLab Fusion Fx6 Edge Imaging system. MST Data was collected using Monolith NT. 115. Data was organised using Excel (Microsoft 365). Needle Pairwise Sequence Alignment tool by EMBOSS Program was used for sequence alignment (Supplementary Fig 1).
Data analysis	MO.Affinity Analysis (version 2.3) was used to analyse the MST experiments. RMSD plots were prepared using QtGrace (v0.2.6). All other plots and statistical analyses were performed using GraphPad Prism 8. ImageJ (version 1.53t) was used to analyse Western blot data.

For manuscripts utilizing custom algorithms or software that are central to the research but not yet described in published literature, software must be made available to editors and reviewers. We strongly encourage code deposition in a community repository (e.g. GitHub). See the Nature Portfolio guidelines for submitting code & software for further information.

Data

Policy information about availability of data

All manuscripts must include a data availability statement. This statement should provide the following information, where applicable:

- Accession codes, unique identifiers, or web links for publicly available datasets
- A description of any restrictions on data availability
- For clinical datasets or third party data, please ensure that the statement adheres to our policy

Source data is provided with the manuscript. Publicly available data referenced: UniProt: O95865; PDB ID:2JAI; NCBI Reference Sequence: NM_012137.4, NM_001303007.2, NP_036269.1, and NP_001289936.1. Source data was provided with the manuscript.

Human research participants

Policy information about studies involving human research participants and Sex and Gender in Research.

Reporting on sex and gender	N/A
Population characteristics	N/A
Recruitment	N/A
Ethics oversight	N/A

Note that full information on the approval of the study protocol must also be provided in the manuscript.

Field-specific reporting

Please select the one below that is the best fit for your research. If you are not sure, read the appropriate sections before making your selection.

[x] Life sciences [ ] Behavioural & social sciences [ ] Ecological, evolutionary & environmental sciences

For a reference copy of the document with all sections, see nature.com/documents/nr-reporting-summary-flat.pdf

Life sciences study design

All studies must disclose on these points even when the disclosure is negative.

Sample size	Recombinant protein experiments were set at min n=2, while activity assay for the in vitro experiments and animal experiments were set at min n=2 and at n=5, respectively, based on the effect size from the pilot data calculated using G-power program (v3.1.9.7).
Data exclusions	Outliers determined by Grubbs test (Figure 6) and ROUT (Figure 8) were removed during data analysis.
Replication	Animal data (n=5) were not replicated as they fit the effect size of the pilot data and to minimize the use of animals for the experiment. MST experiments using recombinant protein (n=3) were not repeated. Recombinant protein enzymatic activity assay were repeated at least twice for each sample, with n=6 or n=9 for the specified samples . Hek293T experiments were repeated at least twice for the activity assay (n=3 for Hek293T DDAH1 Flag, n=2 for Hek293T DDAH2 Flag) and three times for the mRNA expression analysis. MDA-MB-231 activity assay were performed at least three times with a minimum of n=9 and maximum of n=15 for the specified clones while the mRNA expression analysis was replicated 4 times. HUVEC in vitro experiments were performed trice for n=3.
Randomization	Randomization was not relevant to the study as all groups were treated the same.
Blinding	Blinding was not relevant to the study. In order to avoid sampling errors, samples were grouped according to the specific genotype (mutant or wild type). This prevented false analysis of the data which could have occurred if blinding was performed.

Reporting for specific materials, systems and methods

We require information from authors about some types of materials, experimental systems and methods used in many studies. Here, indicate whether each material, system or method listed is relevant to your study. If you are not sure if a list item applies to your research, read the appropriate section before selecting a response.

Materials & experimental systems

n/a	Involved in the study
☐	☒ Antibodies
☐	☒ Eukaryotic cell lines
☒	☐ Palaeontology and archaeology
☐	☒ Animals and other organisms
☒	☐ Clinical data
☒	☐ Dual use research of concern

Methods

n/a	Involved in the study
☒	☐ ChIP-seq
☒	☐ Flow cytometry
☒	☐ MRI-based neuroimaging

Antibodies

Antibodies used	anti-DDAH1 (monoclonal antibody (clone 3H10) by Kimoto et al. 1995; ThermoFisher Scientific PA5-52278 Lot:45560 [1:1000]; Abcam ab180599 [1:1000]), anti-DDAH2 (Abcam ab184166 Lot:GR156739-6 [1:1000]; Abcam ab232694 Lot:GR3439054-1 [1:1000]; St John's Laboratory STJ28540 lot:8540210000101 [1:1000]; Proteintech 14966-1-AP [1:1000]), anti-tubulin (Sigma Aldrich T5168 [1:5000]), anti-beta-actin (Cell Signaling Technology 3700S [1:1000]), anti-beta-actin HRP (Abcam ab499900 [1:30000]).
Validation	Antibodies were validated by the manufacturers by Western blot on human cell lines or mice tissue based on the approximate size of the protein or recombinant human protein. Mutant human cell line and knockout mice tissues also validated the antibodies, as shown in the data.

Eukaryotic cell lines

Policy information about cell lines and Sex and Gender in Research

Cell line source(s)	Human embryonic kidney cell line (HEK293T, #CRL-3216) and human triple negative breast cancer cell line (MDA-MB-231, #CRM-HTB-26) from American Type Culture Condition (ATCC); Human umbilical vein endothelial cell lines (HUVEC, #C2517) from LONZA (Single female donor).
Authentication	Authenticated cell lines were purchased from ATCC and LONZA. Cell morphology was confirmed upon use. No further authentication was performed.
Mycoplasma contamination	The cell lines were not tested for mycoplasma.
Commonly misidentified lines (See ICLAC register)	No ICLAC listed cell lines were used.

Animals and other research organisms

Policy information about studies involving animals; ARRIVE guidelines recommended for reporting animal research, and Sex and Gender in Research

Laboratory animals	The animals were housed in a 12-hour light dark cycle (lights switched on at 06:00) with food and water ad libitum. The animals were housed in rooms set to an ambient temperature of 22-24°C and humidity of 45–46%. Global Ddah1 deficient C57Bl/6J mice were developed by Hu et. al. 2011 (ref 35) (3m+2f) ; Global Ddah2 deficient mice line was purchased from Taconic (Model #TF0168) (wild type: 3m+2f; knockout: 4m+1f). Animals were sacrificed at ages 18-22 weeks.
Wild animals	No wild animals were used in the study.
Reporting on sex	Groups were made of mixed sex animals. Sex was not considered in the study to avoid bias due to a specific sex group.
Field-collected samples	No field-collected samples were used.
Ethics oversight	Animals were humanely sacrificed according to protocols approved by the animal welfare committee of Technische Universität Dresden. Organ collection from mice for the experiments was performed in accordance to the ethical permissions DD25-5131/530/11 and 24-9168.24-1/2014-2.

Note that full information on the approval of the study protocol must also be provided in the manuscript.
