# Supplementary material for: A multicentric consortium study demonstrates that dimethylarginine dimethylaminohydrolase 2 is not a dimethylarginine dimethylaminohydrolase
Source: Nat Commun. 2023 Jun 9;14:3392. doi: 10.1038/s41467-023-38467-9 (PMC10256801; doi:10.1038/s41467-023-38467-9)
Supplement: Supplementary file 9 — Source Data [file 41467_2023_38467_MOESM9_ESM.zip › Source Data/File 11_Western blot and agarose gel raw files.pptx]

## Slide 1
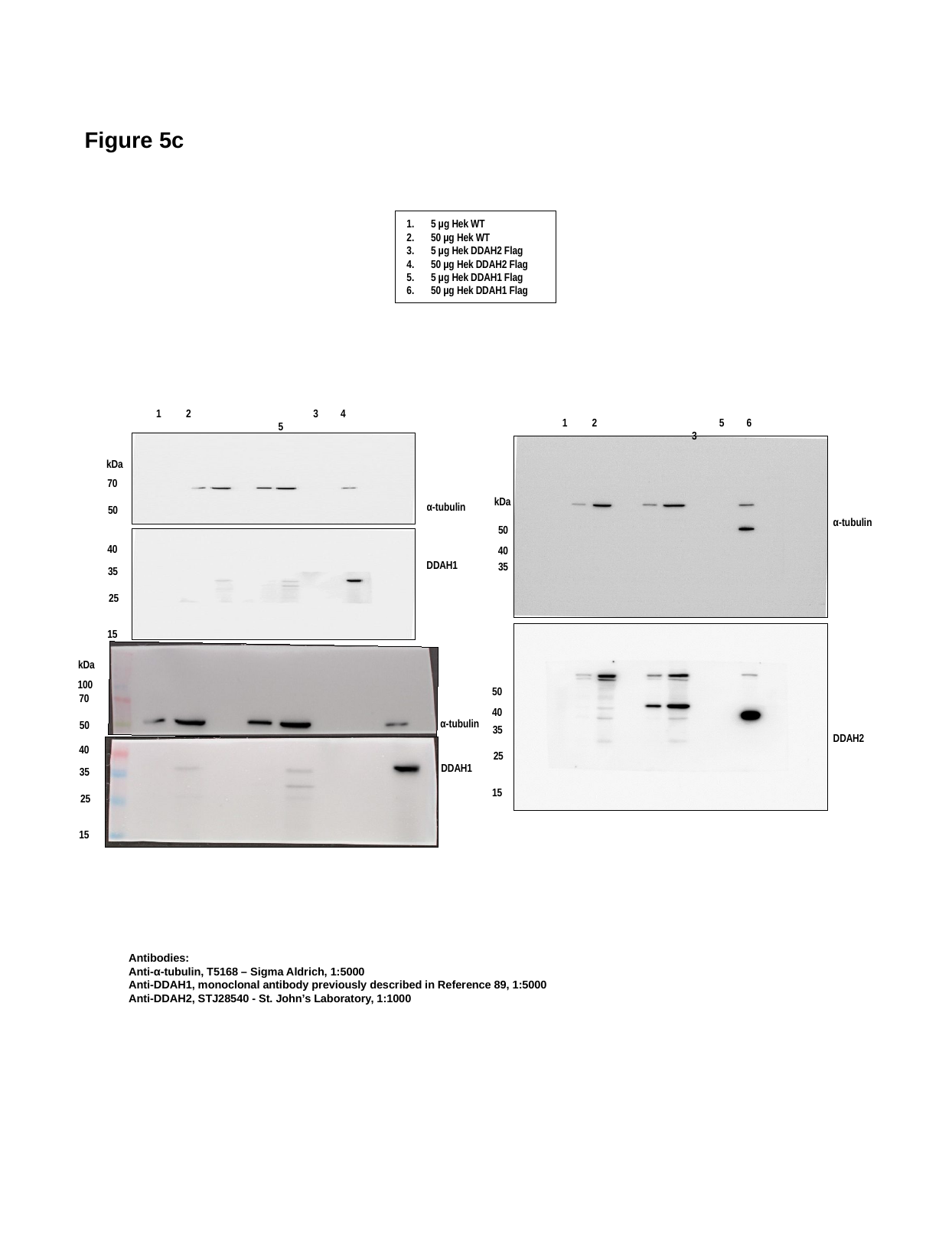

Figure 5c
5 µg Hek WT
50 µg Hek WT
5 µg Hek DDAH2 Flag
50 µg Hek DDAH2 Flag
5 µg Hek DDAH1 Flag
50 µg Hek DDAH1 Flag
1 2	 3 4		 5
α-tubulin
DDAH1
DDAH1
α-tubulin
kDa
70
50
40
35
25
15
kDa
100
70
50
40
35
25
15
1 2	 5 6		 3
α-tubulin
DDAH2
50
40
35
50
40
35
25
15
kDa
Antibodies:
Anti-α-tubulin, T5168 – Sigma Aldrich, 1:5000
Anti-DDAH1, monoclonal antibody previously described in Reference 89, 1:5000
Anti-DDAH2, STJ28540 - St. John’s Laboratory, 1:1000

## Slide 2
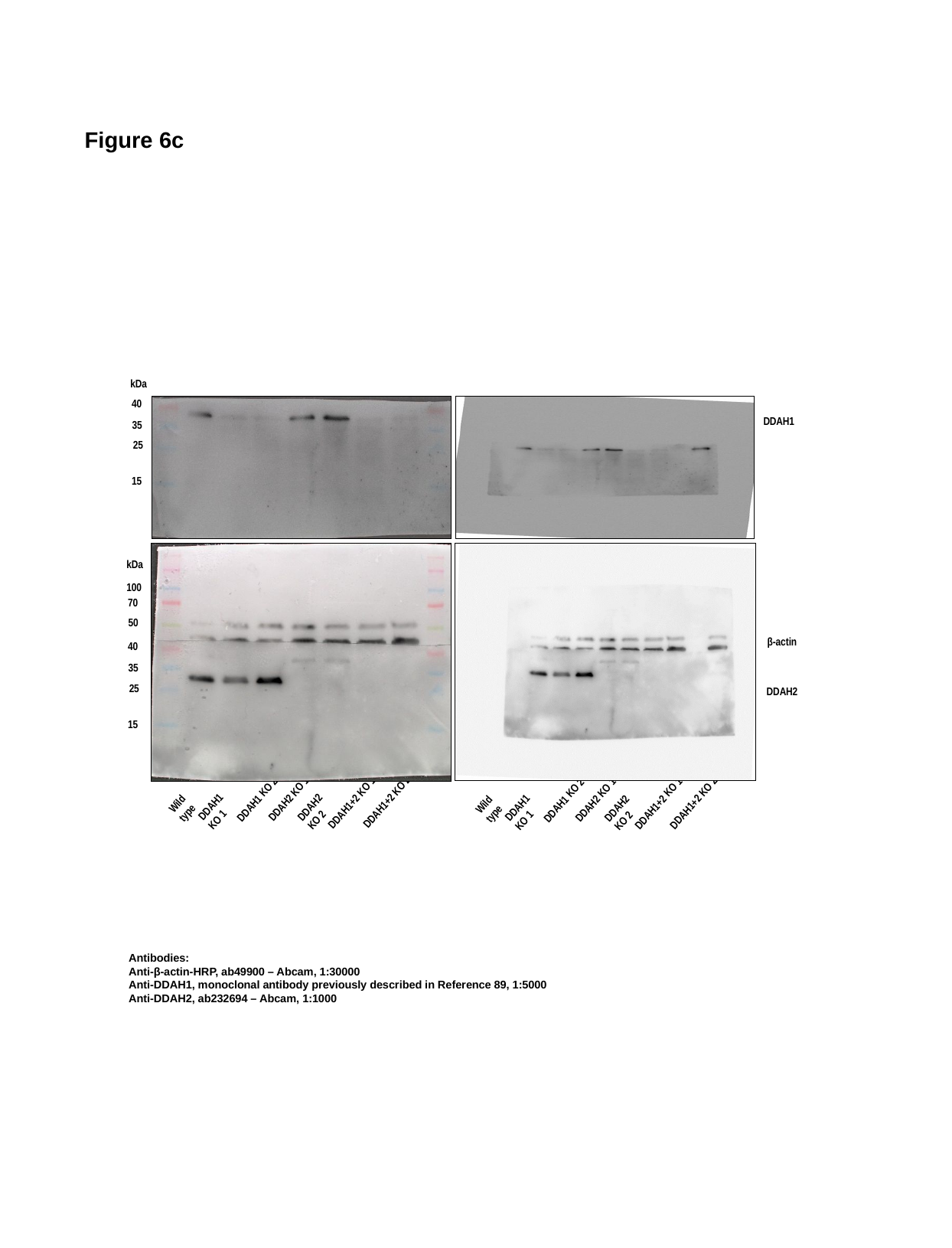

Figure 6c
kDa
40
35
25
15
DDAH2 KO 1
DDAH1 KO 2
DDAH1+2 KO 2
DDAH1+2 KO 1
DDAH1 KO 1
DDAH2 KO 2
Wild type
DDAH2 KO 1
DDAH1 KO 2
DDAH1+2 KO 2
DDAH1+2 KO 1
DDAH1 KO 1
DDAH2 KO 2
Wild type
DDAH1
β-actin
DDAH2
kDa
100
70
50
40
35
25
15
Antibodies:
Anti-β-actin-HRP, ab49900 – Abcam, 1:30000
Anti-DDAH1, monoclonal antibody previously described in Reference 89, 1:5000
Anti-DDAH2, ab232694 – Abcam, 1:1000

## Slide 3
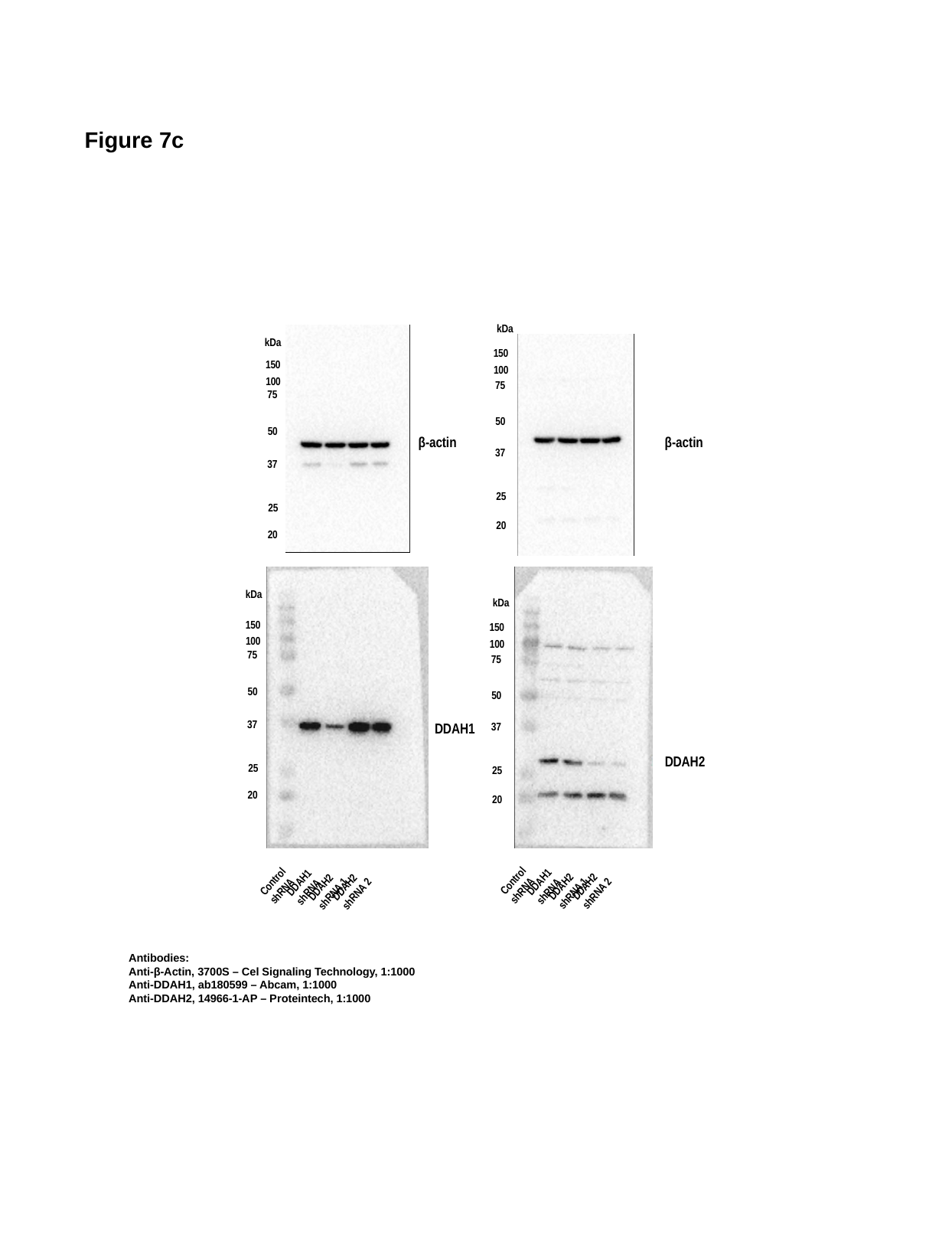

Figure 7c
kDa
150
100
75
50
37
25
20
β-actin
DDAH2
Control shRNA
DDAH1 shRNA
DDAH2 shRNA 1
DDAH2 shRNA 2
kDa
150
100
75
50
37
25
20
β-actin
DDAH1
kDa
150
100
75
50
37
25
20
kDa
150
100
75
50
37
25
20
Control shRNA
DDAH1 shRNA
DDAH2 shRNA 1
DDAH2 shRNA 2
Antibodies:
Anti-β-Actin, 3700S – Cel Signaling Technology, 1:1000
Anti-DDAH1, ab180599 – Abcam, 1:1000
Anti-DDAH2, 14966-1-AP – Proteintech, 1:1000

## Slide 4
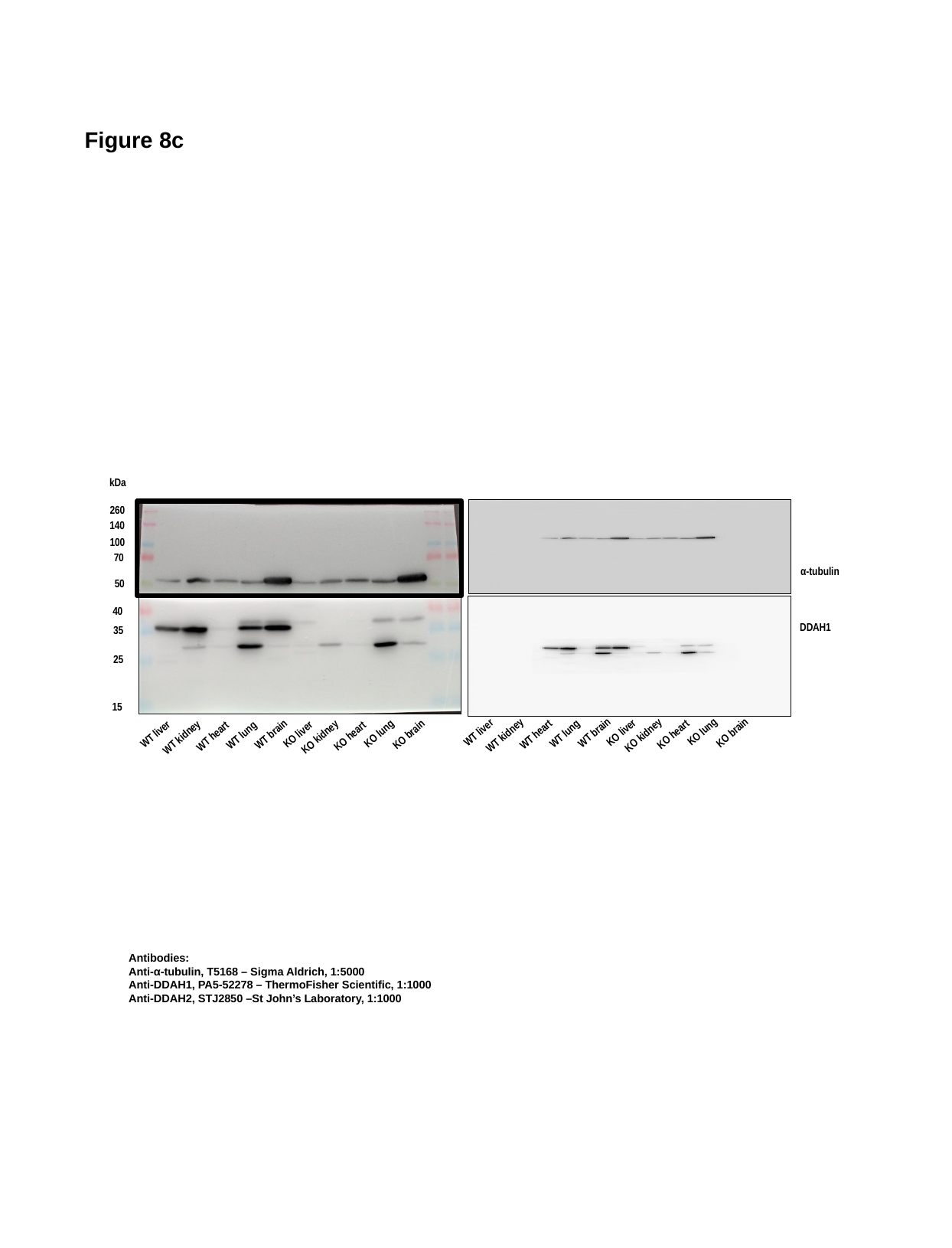

Figure 8c
kDa
260
140
100
70
50
40
15
35
25
α-tubulin
DDAH1
WT liver
WT brain
KO lung
KO brain
KO liver
WT lung
KO heart
WT heart
WT kidney
KO kidney
WT liver
WT brain
KO lung
KO brain
KO liver
WT lung
KO heart
WT heart
WT kidney
KO kidney
DDAH2
Antibodies:
Anti-α-tubulin, T5168 – Sigma Aldrich, 1:5000
Anti-DDAH1, PA5-52278 – ThermoFisher Scientific, 1:1000
Anti-DDAH2, STJ2850 –St John’s Laboratory, 1:1000

## Slide 5
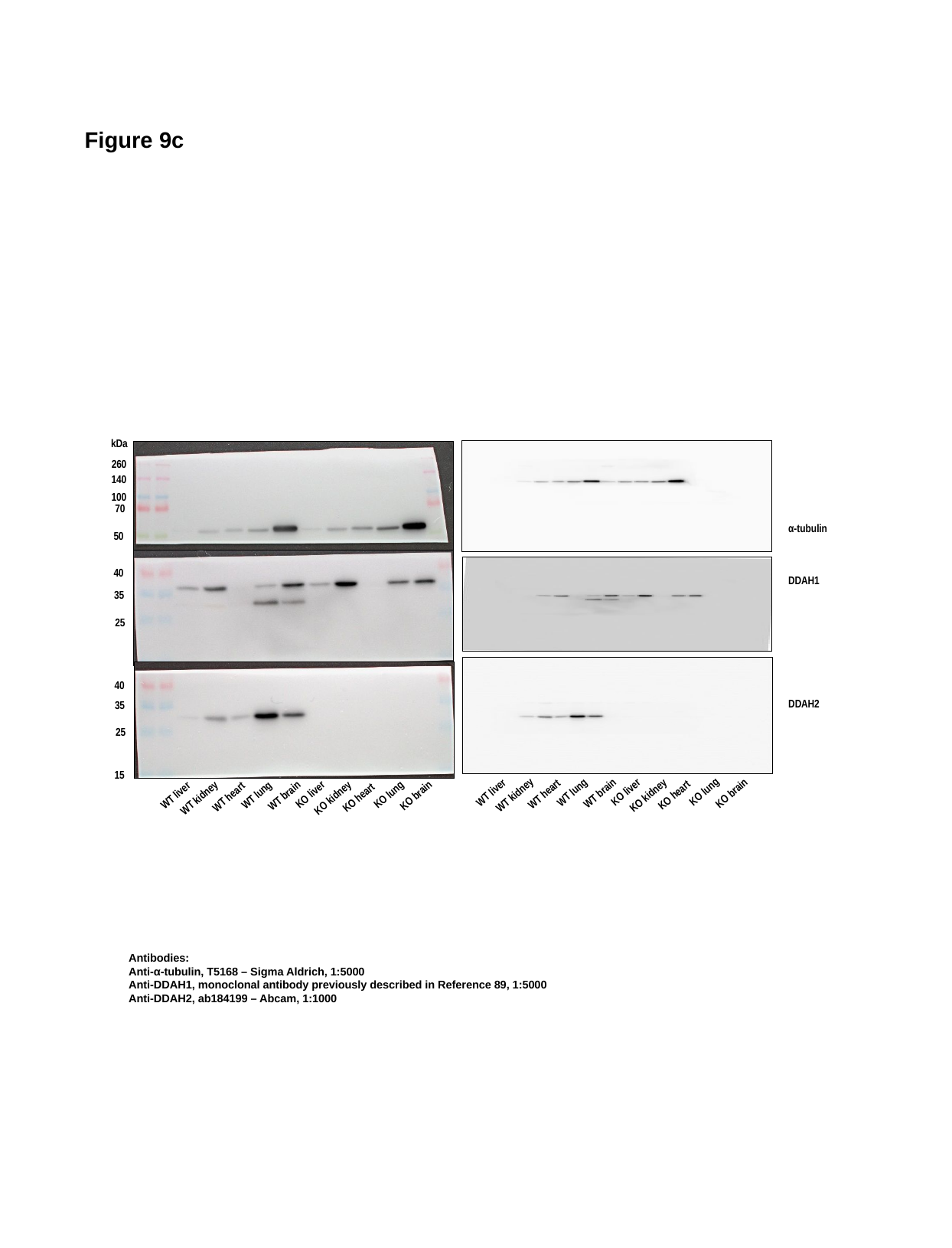

Figure 9c
kDa
α-tubulin
DDAH2
DDAH1
WT liver
WT lung
WT brain
WT heart
WT kidney
KO lung
KO liver
KO brain
KO heart
KO kidney
WT liver
WT lung
WT brain
WT heart
WT kidney
KO lung
KO liver
KO brain
KO heart
KO kidney
260
140
100
70
50
40
35
25
40
35
25
15
Antibodies:
Anti-α-tubulin, T5168 – Sigma Aldrich, 1:5000
Anti-DDAH1, monoclonal antibody previously described in Reference 89, 1:5000
Anti-DDAH2, ab184199 – Abcam, 1:1000

## Slide 6
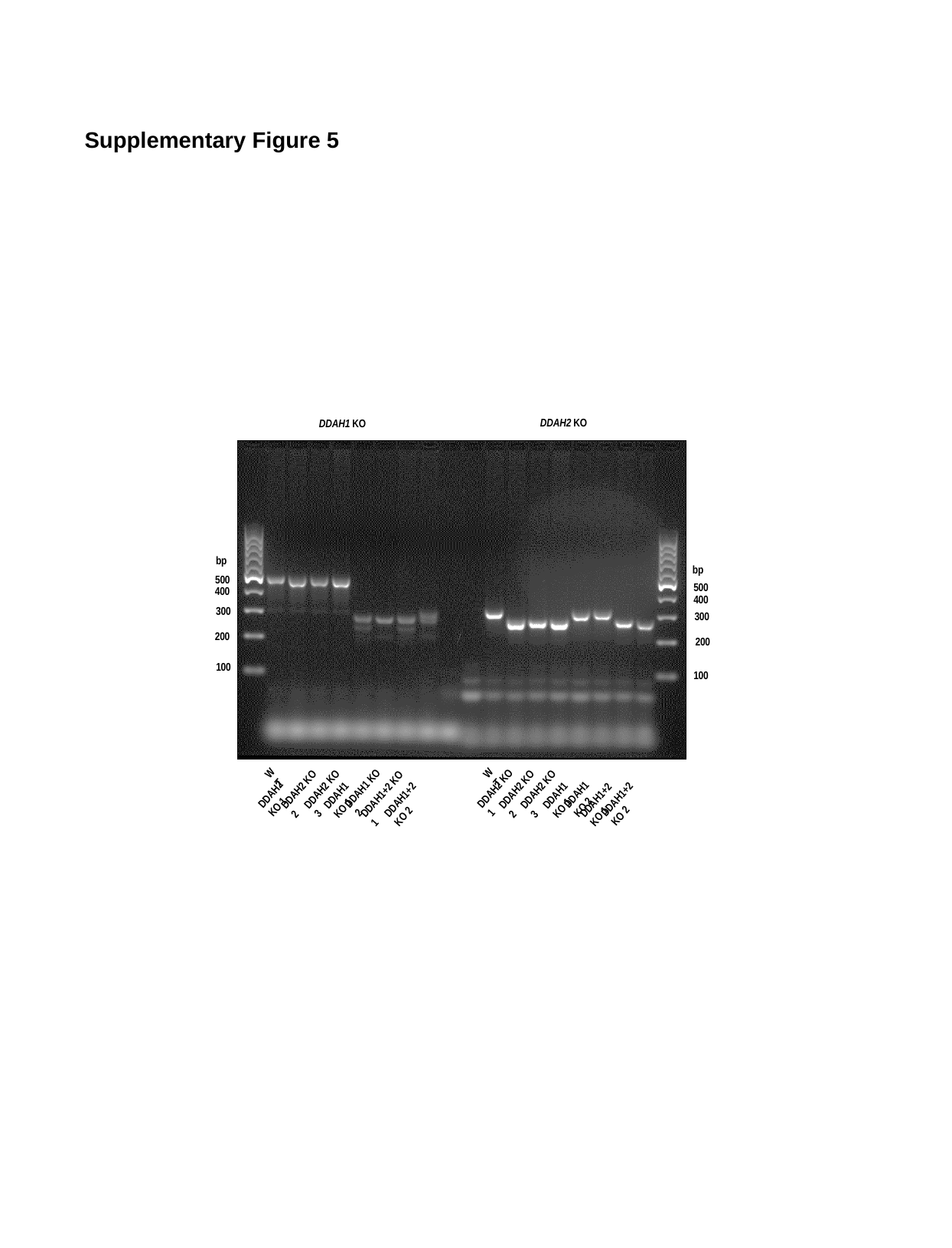

Supplementary Figure 5
DDAH2 KO
DDAH1 KO
500
300
400
200
100
500
400
300
200
100
DDAH2 KO 2
DDAH2 KO 1
DDAH1+2 KO 2
DDAH1 KO 2
DDAH1 KO 1
DDAH1+2 KO 1
WT
DDAH2 KO 3
DDAH2 KO 2
DDAH1 KO 2
DDAH1+2 KO 1
DDAH1 KO 1
DDAH1+2 KO 2
DDAH2 KO 1
WT
DDAH2 KO 3
bp
bp
