# Supplementary material for: A multicentric consortium study demonstrates that dimethylarginine dimethylaminohydrolase 2 is not a dimethylarginine dimethylaminohydrolase
Source: Nat Commun. 2023 Jun 9;14:3392. doi: 10.1038/s41467-023-38467-9 (PMC10256801; doi:10.1038/s41467-023-38467-9)
Supplement: Supplementary file 9 — Source Data [file 41467_2023_38467_MOESM9_ESM.zip › Source Data/Source Data List.docx]

**Source Data List**

| File Number | File Name | Reference Figure/Table |
| --- | --- | --- |
| File 1 | DDAH-ADMA Molecular dynamics simulation | Figure 2e-g, Supplementary Table 1 |
| File 2 | MST Dose Response Raw Data Grouped | Figure 3 |
| File 3 | Recombinant DDAH protein data | Figure 4 |
| File 4 | Hek293T cells overexpressing DDAH1 and DDAH2 | Figure 5a-b, d |
| File 5 | DDAH activity assay of lysate from wild type and mutant MDA-MB-231 cell lines | Figure 6a-b, d |
| File 6 | DDAH activity assay of lysate from wild type and mutant HUVEC | Figure 7a-b, d |
| File 7 | DDAH activity assay of lysate from wild type and Ddah1 mutant mice | Figure 8a-b, d |
| File 8 | DDAH activity assay of lysate from wild type and Ddah2 mutant mice | Figure 9a-b, d |
| File 9 | Intrinsic fluorescence spectra of recombinant DDAH proteins | Supplementary Figure 3 |
| File 10 | DDAH2 Model A (SwissModel)-ADMA, Pose A – C, Molecular dynamics simulation 1- 3 | Supplementary Figure 5b-d |
| File 11 | Western blot and agarose gel raw images | Figures 5c, 6c, 7c, 8c, 9c, Supplementary Figure 4 |
| File 12.1-12.3 | ADMA Input coordinate, ADMA output optimized coordinate, and ADMA Topology | ADMA configurations in Figures 2e-g, Supplementary Figures 6b-d |
| File 13.1-13.10 | DDAH1-X-ray MDS Initial and Final configurations | Figure 2e |
| File 14.1-14.10 | DDAH2-ModelA MDS Initial and Final Configurations | Figure 2f |
| File 15.1-15.10 | DDAH2-ModelB MDS Initial and Final Configurations | Figure 2g |
| File 16.1-16.6 | DDAH2-ModelA MDS PoseA Initial and Final Configurations | Supplementary Figure 6b |
| File 17.1-17.6 | DDAH2-ModelA MDS PoseB Initial and Final Configurations | Supplementary Figure 6c |
| File 18.1-18.6 | DDAH2-ModelA MDS PoseC Initial and Final Configurations | Supplementary Figure 6d |
